# On the Use of Paper Sludge as Filler in Biocomposites for Injection Moulding

**DOI:** 10.3390/ma14102688

**Published:** 2021-05-20

**Authors:** Vito Gigante, Patrizia Cinelli, Marco Sandroni, Roberto D’ambrosio, Andrea Lazzeri, Maurizia Seggiani

**Affiliations:** 1 Department of Civil and Industrial Engineering, University of Pisa, Largo Lucio Lazzarino 2, 56122 Pisa, Italy; vito.gigante@dici.unipi.it (V.G.); marco.sandroni@unipi.it (M.S.); roberto.dambrosio@ing.unipi.it (R.D.); andrea.lazzeri@unipi.it (A.L.); 2 Department Consorzio Interuniversitario Nazionale per la Scienza e Tecnologia dei Materiali (INSTM), Via Giusti 9, 50121 Florence, Italy

**Keywords:** biocomposites, paper sludge, waste, extrusion, mechanical properties, phytotoxicity

## Abstract

The potential use of paper sludge (PS) as filler in the production of bio-composites based on poly lactic acid (PLA) and polybutylene adipate terephthalate (PBAT) was investigated. PS/PLA/PBAT composites, with addition of acetyl tributyl citrate (ATBC) as biobased plasticizer, were produced with PS loadings up to 30 wt.% by twin-screw extrusion followed by injection moulding. The composites were characterized by rheological measurements, thermogravimetric analysis (TGA), differential scanning calorimetry (DSC) and mechanical tests (tensile and impact resistance) to study the effect of PS on the processability, thermal stability, crystallinity and mechanical performance of polymeric matrix. The optimized composites at higher PS content were successfully processed to produce pots for horticulture and, in view of this application, preliminary phytotoxicity tests were conducted using the germination test on *Lepidium sativum* L. seeds. Results revealed that developed composites up to 30 wt.% PS had good processability by extrusion and injection moulding showing that PS is a potential substitute of calcium carbonate as filler in the production of bio-composites, and the absence of phytotoxic effects showed the possibility of their use in the production of pots/items for applications in floriculture and/or horticulture.

## 1. Introduction

New environmental and circular economy politics demand for the development of 100% bio-based closed loops maximizing the re-usage of each component present into the waste processing streams of the industries [1,2]. Therefore, sustainable strategies are necessary to deal with the large amounts of industrial by-products finding viable commercial applications.

Within this framework, the European paper and board industry accounted for more than 14 million tons of waste in 2019, where 54.5% of them derived from recycled paper production [3]. The main goal of the recycling process is to reclaim cellulose fibers while retaining paper quality both in terms of mechanical and optical properties.

In order to reuse those fibers, all the contaminants present have to be removed thus leading to the onset of different kinds of sludge which need to be properly disposed. Accordingly, the tissue paper production from recycled paper generates paper sludge (PS) up to 150 kg dry solids/tons of product [4].

The common disposal strategies for PS concern the landfilling and/or incineration, but several potential re-uses deriving from its composition can be explored [5,6]. PS, on dry basis, is mainly composed of short cellulose fibers and inorganic fillers as calcium carbonate (CaCO_3_) (added along the production stage of recovered paper to achieve an improvement in its mechanical and optical properties) [6], small amount of heavy metals and functional and process chemicals.

Among the several valorization alternatives proposed for PS, biochar derived from its pyrolysis was employed in soil remediation contaminated by heavy metals due to its high contents of cellulose and reduced toxic components [7,8]. Moreover, PS can be converted into high-grade fuel and chemicals by different thermochemical approaches to provide energy [9]. Several studies have been reported also on the use of PS as filler in the manufacture of building materials (e.g., cements, bricks, concrete) [10,11].

Due to its high content of CaCO_3_, many studies have carried out on the use of PS as filler or reinforcing in composites with thermoplastic polymers as polypropylene (PP) [12,13], high density polyethylene (HDPE) [14,15,16], and recycled HDPE [17].

In this context one of the major concerns to reach a practical application is to guarantee a sufficient interfacial filler/polymer matrix adhesion to improve the stress transfer between the two components. The polar-hydrophilic nature of paper sludge fibers with respect to the typically nonpolar-hydrophobic thermoplastic matrix needs the application of specific approaches aimed at improving the fibre-matrix interfacial bonding. This issue was addressed by grafting polymer matrix with hydrophilic functional group [18] or by using compatibilizers that bond the non-polar polymer matrix to the polar fibers [19].

The potential use of PS as filler in thermoplastic composites was extended to bioplastics as poly lactic acid (PLA) and polybutylene adipate terephthalate (PBAT) in the present work. PLA is one of the most studied renewable polymers, fully biobased and compostable. It represents a sustainable alternative to petro-derived plastics for a large variety of applications, ranging from the industrial to biomedical field. However, its brittleness and its poor barrier properties restrict its range of application [20]. To overcome these drawbacks, several strategies have been proposed as modification of the chemical structure of PLA chains by co-polymerization of lactide with different monomers [21]; and formulation of PLA-based blends by melt blending [22]. Among them, blending is a much more practical and cost-effective method compared to co-polymerization and, for these reasons, is the more frequently used method at industrial level. In this case, by adjusting the weight ratio between the selected polymers, tuning of mechanical and thermal properties of the blends can be achieved. Several binary and ternary blends with ductile polymers to improve PLA reduced toughness and flexibility have been reported in literature [23]. Among them, PBAT blended with PLA at percentages from 10 to 25 wt.% shows to be a valid toughening agent leading to a significant improvement of the elongation at break and Charpy impact resistance [24]. Ternary plasticized blends PLA/PBAT coupled with a polyolefin elastomer grafted with glycidyl methacrylate (POE-g-GMA) with addition (10 wt.%) of a biobased plasticizer were carried out to achieve an additional increase of the impact properties and tensile flexibility [25]. In particular, two different plasticizers were evaluated, one not reactive as acetyl tributyl citrate (ATBC) and one reactive as glycidyl ether (EJ-400), to further increase the polymers mobility, connected to the improvement of the elongation at break maintaining an adequate impact resistance.

In this work, PS/PLA/PBAT/ATBC composites were produced with different PS loadings (10, 20 and 30 wt.%) by twin-screw extrusion. The produced composites were characterized by rheological measurements, thermogravimetric analysis (TGA), differential scanning calorimetry (DSC) and mechanical tests (tensile and impact resistance) to study the effect of PS loading on the processability, thermal stability, crystallinity and mechanical performance of polymeric matrix. The optimized composites were processed by injection molding to produce pots for horticulture and, in view of this application, preliminary phytotoxicity tests based on the germination index of *Lepidium sativum* L. seeds were conducted using eluates of PS and PS-based composites.

## 2. Materials and Methods

### 2.1. Materials

The materials used for the production of the bio-composites were the following.

Paper sludge (PS) was provided by Lucart S.p.A. (Lucca, Italy) in form of amorphous grey granular material with an apparent density of 0.97 g/cm^3^.

PLA2003D (extrusion grade) was purchased from NatureWorks (Minnetonka, MN, USA) having a melt flow index (MFI): 6 g/10 min (210 °C, 2.16 kg), nominal average molar mass: 180,000 g/mol, density: 1.24 g/cm^3^. It contains about 4% of D-lactic acid units which lower the melting point and crystallization tendency, improving the processability during the melt extrusion.

PBAT Ecoflex^®^ C1200 was purchased from BASF (Ludwigshafen am Rhein, Germany). It is a biodegradable, random aliphatic-aromatic co-polyester based on the monomers 1.4-butanediol, adipic acid and terephthalic acid, having an MFI: 4 g/10 min (190 °C, 2.16 kg), nominal average molar mass: 126,000 g/mol, density: 1.26 g/cm^3^.

Acetyl tributyl citrate (ATBC) was purchased from Tecnosintesi S.p.A (Bergamo, Italy); it was used as biobased and biodegradable plasticizer. It is a colourless and odourless liquid having a density of 1.05 g/cm^3^ and a molar mass of 402.5 g/mol.

Calcium carbonate, CaCO_3_, (OMYACARB 1-AV) was purchased from Omya (Avenza/Carrara, Italy) and used as inorganic filler (average particle diameter of 1.6 μm).

Calcium Stearate was purchased from Kemistab (Barcelona, Spain) and used as demoulding lubricant. It is a white powder with an acid value of 2% and calcium content of 7 wt.%.

### 2.2. Methods

#### 2.2.1. Paper Sludge Pre-Treatment

PS was initially conditioned in a ventilated oven at 55 °C for 24 h, in order to make it sufficiently dry for the subsequent pulverization, carried out in a Fritsch pulveriser at 20,000 rpm with a 2 mm grid.

The resultant powder was then dried in an oven at 105 °C for 24 h. After drying, the sample was subjected to a second pass in the pulveriser. A small part of the obtained powder (below 2 mm) was used for the subsequent characterizations and the rest was used for the production of the composites.

#### 2.2.2. Composites Production

In choosing the composition of the biocomposites to be studied, previous experiences on binary and ternary blends based on PLA, PBAT and ATBC were taken into account [24,25]. Ratio PLA/PBAT of 3.5 was used with the addition of ATBC as plasticizer to increase the ductility of the composite and counteract the expected fragility due to the addition of filler.

Grinded PS (with or without calcium carbonate) was fed by a gravimetric side feeder in a COMAC EBC 25 HT (Milan, Italy) co-rotating twin-screw extruder with L/D = 44 together with PLA/PBAT granules (fed by the main feeder) and the liquid plasticizer fed by injection. The composition of the different produced composites is shown in Table 1. The extruded filaments were cooled in a water tank, blown with an air fan and then pelletized into 1 mm diameter granules. The granules were dried in a PIOVAN DP 604-615 dryer (Venice, Italy) at 50 °C for 24 h and, then, used for the subsequent characterizations and processing by injection molding.

As shown in Table 1, in the Matrix1 and Matrix1c the ratio PLA/(PBAT + ATBC) was always kept constant at 2.3 and in the Matrix1c, calcium carbonate was added as inorganic filler to evaluate the effect of mixture PS/CaCO_3_ and to compare the effect of PS to that of CaCO_3_. In all the formulations 1 wt.% (respect to the total of filler) of calcium stearate has been added to the filler in order to improve its flow-ability and to achieve a more homogeneous distribution within the polymer matrix [26].

#### 2.2.3. Lab-Scale Injection Moulding

**Figure 1:**
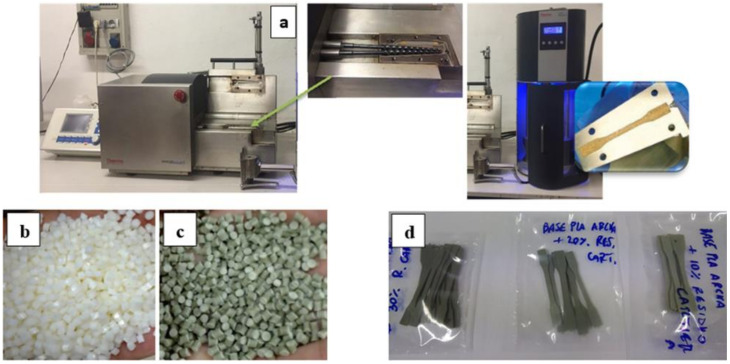
(**a**) Haake MiniLab mini-extruder equipped with Haake MiniJet II injection moulding (**b**) granules of Matrix1; (**c**) granules of Matrix1_20 (**d**) dog bone specimens containing 10, 20 and 30 % wt. of the PS (Matrix1 series).

Using the Haake Minilab-MiniJet microcompounder (Thermofisher, Karlsruhe, Germany) (Figure 1a), the granules (Figure 1b,c) at different composition were used to produce “dog bone” specimens (Haake III Type 557-2290, size: 25 × 5 × 1.5 mm^3^) for the tensile tests (Figure 1d) and specimens (ISO179:2010 [27], size: 80 × 10 × 4 mm^3^) for the Charpy impact tests.

The operating conditions in minilab and Minijet were set as follows: injection chamber temperature: 190 °C, screw rotation speed: 100 rpm, mould temperature: 43–45 °C, injection pressure: 770 bar, pressure application time: 9 s, post pressure: 400 bar, post-pressure time: 15 s.

### 2.3. PS and Composite Characterizations

Powdered PS was analysed by scanning electron microscope (SEM) FEI Quanta 450 FEG (ThermoFisher, Waltham, MA, USA) equipped with a large field detector for low kV imaging simultaneous secondary electron (SE). The sample was previously gold sputtered by using a sputter coater Edward S150B.

Thermogravimetric analysis (TGA) was conducted on the grinded PS using a TA Q-500 thermobalance (TA Instruments, Waters LLC, New Castle, DE, USA) to assess the thermal stability of the material and, consequently, the feasibility of its use at extrusion processing temperatures of PLA/PBAT matrix (180–200°C). About 20 mg of sample was heated in N2 from room temperature to 800 °C at 10 °C/min and the weight and rate of weight loss as a function of temperature were recorded.

Differential scanning calorimetric (DSC) analysis was conducted on the Matrix1 series using a Pyris (Perkin Elmer Instrument, Waltham, MA, USA) equipped with a Perkin Elmer IntraCooler 1 as a refrigerating system. The samples were subjected to 1st heating from 20 to 160 °C at a rate of 10 °C/min, followed by cooling from 160 to 20 °C at a rate of 20 °C/min and 2nd heating from 20–200 °C at a rate of 10 °C/min.

The crystallinity percentage (*X_cc_*) of PLA in the binary blends was calculated as follow Equation (1):

(1) $$X_{cc\left(PLA\right)}=\frac{\Delta H_{m\left(PLA\right)}-\Delta H_{cc\left(PLA\right)}}{\Delta H{{}^{\circ}}_{m\left(PLA\right)}wt_{PLA}}\times 100$$

where Δ*H*_*m*(*PLA*)_ is the PLA melting enthalpy, Δ*H*_*cc*(*PLA*)_ is the PLA cold crystallization enthalpy, and Δ*H*°_*m*(*PLA*)_ is the melting enthalpy of 100% crystalline PLA, taken equal to 93 J/g [28], *wt_PLA_* is the total mass fraction of PLA in the blends. As a reference with pure PLA, a sample injection moulded dog-bone specimen of pure PLA was also thermal characterized.

Quasi-static tensile tests were carried out at room temperature on dog-bone specimens in accordance with ASTM D 638 with a crosshead speed of 10 mm/min, by an Instron 5500R universal testing machine (Canton, MA, USA) equipped with a 1 kN load cell, and interfaced with a computer running MERLIN Instron software 2.0 (Instron, Canton, MA, USA).

Charpy impact tests were performed at room temperature on 2-mm V-notched specimens using a 15 J Charpy pendulum (CEAST 9050, Instron, Canton, MA, USA) following the standard method ISO 179:2000.

For each mechanical test, at least five replicates were carried out.

Rheological analysis of the Matrix1 and its composites was carried out using an Anton Paar MCR 92 rheometer (Anton Paar, Graz, Austria), with parallel-plate setup (25 mm diameter, 1 mm gap), in the temperature range of 160–200 °C and at frequencies in the range 0.314–28 rad/s. All measurements were conducted under 0.2% strain to ensure that they were done in the linear viscoelastic region. The storage modulus G′, the loss modulus G″ and complex viscosity η* were recorded as a function of angular frequency ω. All the samples were dried before testing and three replicates were carried out for each sample.

The optimized composites were used to produce pots for horticulture by injection moulding in order to validate their industrial processability.

Finally, in view of the potential use of PS-based composites for the production of items for applications in agriculture, a preliminary phytotoxicity test was performed using *Lepidium sativum* L. seeds exposed to eluates from powdered PS and pellets containing 30 wt.% PS (sample Matrix1_30). Powdered PS and pellets of size below 2 mm were subjected to extraction in deionised water for 24 h (1:10 g:mL). The extract was then filtered through 0.45 µm filter membranes and aliquots of 2.5 mL of eluate, as it is (100%) and diluted with deionised water (50%), were placed in Petri dishes (diameter of 90 mm) containing a Whatman filter paper disc. In each capsule, 10 seeds of *L. sativum* were added. The seeds inside the capsules were incubated in a thermostatic chamber at a temperature of 24–26 °C for 72 h [28].

**Figure 2:**
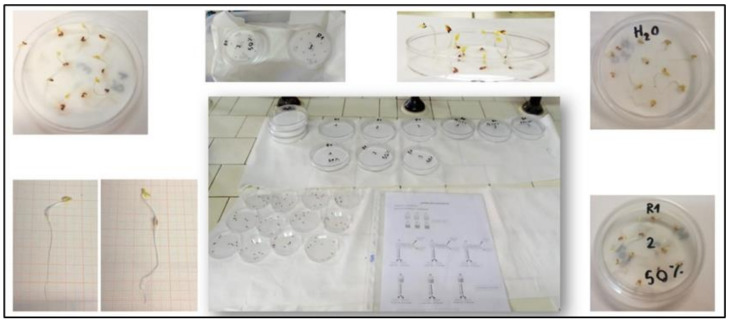
Set-up of the *L. sativum* seed germination test and germinated seeds after 72 h.

The germination index (GI) of *Lepidium sativum* L. seeds was calculated by the Equation (2) [29]:

(2) $$IG\left(\%\right)=\frac{Gc\cdot Lc}{Gt\cdot Lt}\times 100$$

where: *Gc* and *Gt* is number of germinated seeds in the sample and with the deionised water, respectively; *Lc* and *Lt* is mean root length in the sample and with the deionised water, respectively. Three replicates were performed for each sample (Figure 2).

## 3. Results and Discussion

### 3.1. Morphological Analysis

**Figure 3:**
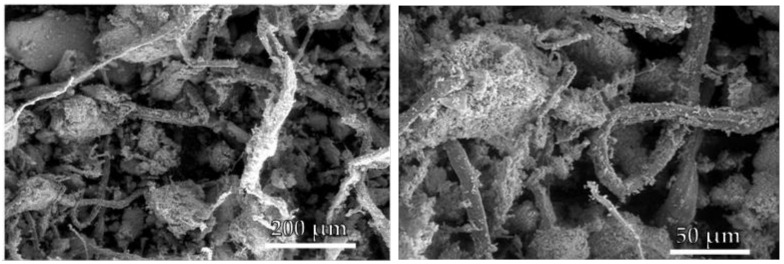
SEM images of pulverised PS at various magnifications: ×500 (**left**) and ×1600 (**right**).

Figure 3 shows SEM images of the grinded-dried PS at various magnifications.

It can be seen irregular, cracked, and short fibres, mixed with aggregates of mineral particles (calcium carbonate). These fibres have length of 200–500 microns with a diameter of around 10 microns (average L/D = 35).

### 3.2. Thermogravimetric Analysis

**Figure 4:**
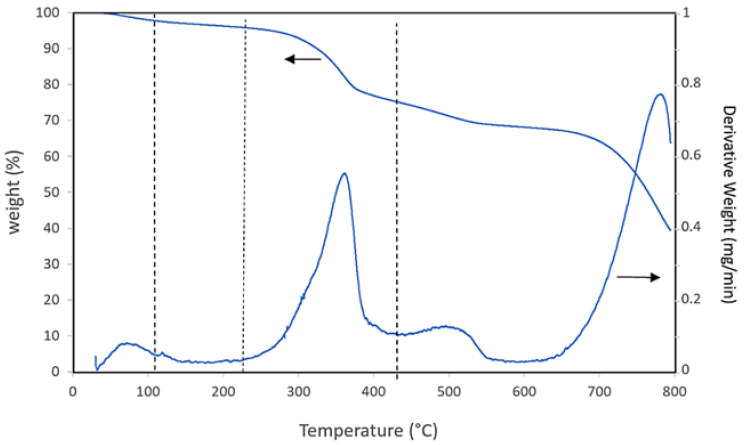
TG and DTG curves of the paper industry residue.

Figure 4 shows the thermogravimetric (TG) and derivate thermogravimetric (DTG) curves of the PS obtained under nitrogen atmosphere as a function of temperature.

As can be seen, the PS has an initial weight loss of about 4% from room temperature to 105 °C due to the moisture adsorbed before the test; up to 220 °C PS is thermally stable and starts to degrade after 220 °C with a weight loss of 16% up to 390 °C, attributable to the decomposition of hemicelluloses and cellulose of fibres [30]. At higher temperatures the decomposition of the organic fraction is completed and the decomposition of the calcium carbonate starts above 600 °C [31]. Thus, the thermal stability of PS up to 220 °C attests its suitability to be processed with PLA/PBAT matrix having extrusion temperatures below these values and, consequently, no significant PS degradation is expected during its processing by extrusion. 

### 3.3. Tensile Tests

**Figure 5:**
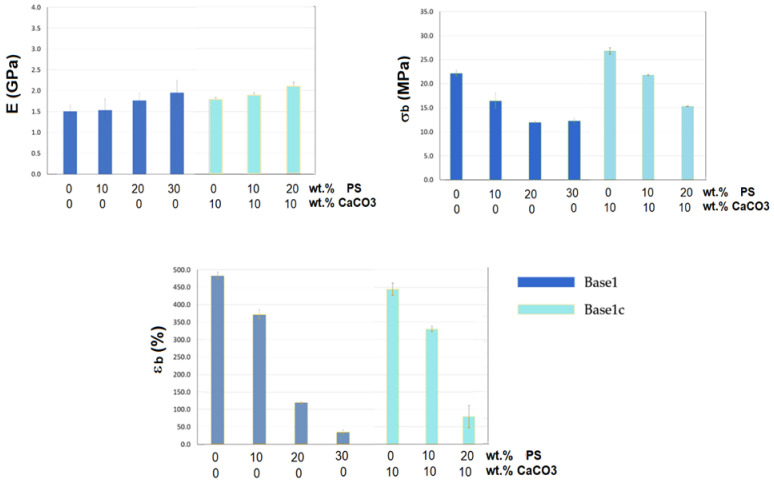
Tensile properties of Matrix1 and Matrix1c composites.

Table 2 shows the effect of the amount and nature of filler (PS or PS/CaCO_3_) on the tensile properties of the various developed composites. In addition, to facilitate the comparison between the various formulations and to better analyze the effect of PS content and the total filler content on the mechanical properties of the PLA/PBAT/ATBC matrix, the results of tensile test are reported in Figure 5.

It can be firstly noticed, in general, that the elastic modulus increased with the addition of filler (PS or mixture PS/CaCO_3_) and a concomitant reduction of the stress at break occurred, more pronounced for the composites with only PS as filler (series Matrix1). Given that the fibres are irregular, cracked, and short, mixed with aggregates of mineral particles, it was expected that there would be a reduction in elongation at break compared to the matrix. The cause is related to the increasing of stress intensification points and defects that lead to specimen failure even at lower breaking load values. On the other hand, it has been observed an increase in stiffness in all the biocomposites.

These results can be attributed to poor interfacial interactions that markedly reduce the stress transfer from the matrix to the fibres, leading to lower tensile strength, as evidenced by several works [32,33,34,35] where the resulting composites exhibited higher elastic modulus but lower tensile strength compared to pure matrix.

Concerning the difference observed between the Matrix1 and Matrix1c composites, with the same total filler content, this can attributable to greater heterogeneity of the PS, consisting of inorganic aggregates and short hemicellulose/cellulose fibres, and larger particle size compared to the used calcium carbonate which leads to a worse distribution of the PS in the matrix with consequent worsening of the breaking load and elongation. In any case, acceptable values of tensile strength are obtained even with 30 wt.% of PS (Matrix1_30); for this reason, the following characterizations were focused on the Matrix1 series where only PS was used as alternative to calcium carbonate, traditionally used as an inorganic filler in polymeric matrices.

### 3.4. Charpy Impact Resistance

The impact strength values (Table 3) obtained for Matrix1 composites showed that the energy absorbed was approximately three times lower than the matrix Matrix1, but higher than the typical values obtained for PLA-based composites containing lignocellulose fibres [36,37]. In any case, the values remain sufficiently high, also up 30 wt.% PS, for their use in the production of items as plant pots.

### 3.5. Rheological Tests

**Figure 6:**
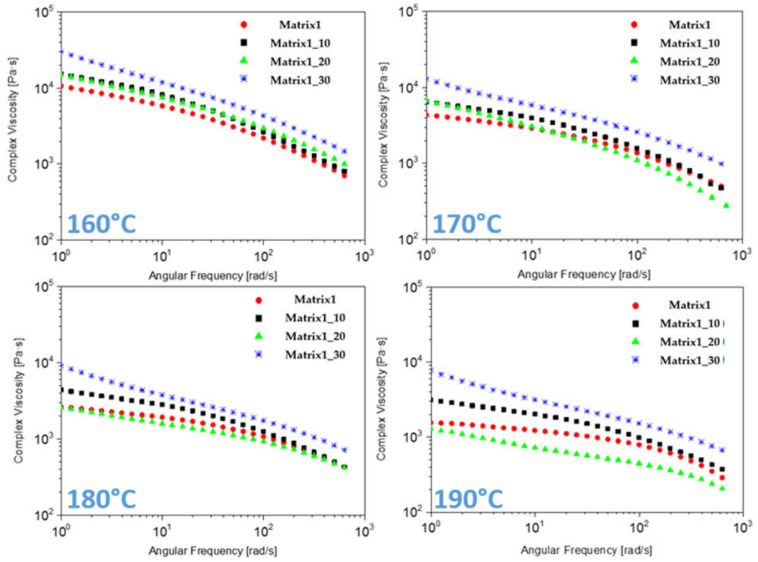
Rotational rheometer measurements at different temperatures.

Figure 6 shows the complex viscosity curves obtained for the Matrix1 composites by rotational rheometer at different temperatures (160–190 °C). All samples showed a shear-thinning as behaviour, without presence of plateau at low frequencies. At the investigated temperatures, the viscosity of Matrix1_30 was the highest, as expected, the viscosity of Matrix1_10 was intermediate while Matrix1 and Matrix1_20 showed the lowest viscosities, often overlapping or very similar. At high frequency, the viscosity of Matrix 1_30 continued to be higher and Matrix1_20 with lower viscosity at temperatures of 170–190 °C; only at 160 °C, the lower the content the lower the viscosity observed. These results can be attributable to the degradation that PS can induce on the PLA by extrusion and subsequent heating during the rheological analysis [38]. In fact, cellulose and residual inner moisture can induce hydrophilic degradation in PLA at high temperatures [39]. So, at high temperatures, up to 20 wt.% PS the effect of degradation of PLA, induced by the hemicellulose/cellulose fibres of PS, determined the increase of fluidity of the composites, whereas at higher PS content (30 wt.%) the filler effect on viscosity overcame the degradation effect. In any case, at high frequencies, all composites showed an adequate viscosity for their process-ability by injection moulding.

### 3.6. DSC Analysis

**Figure 7:**
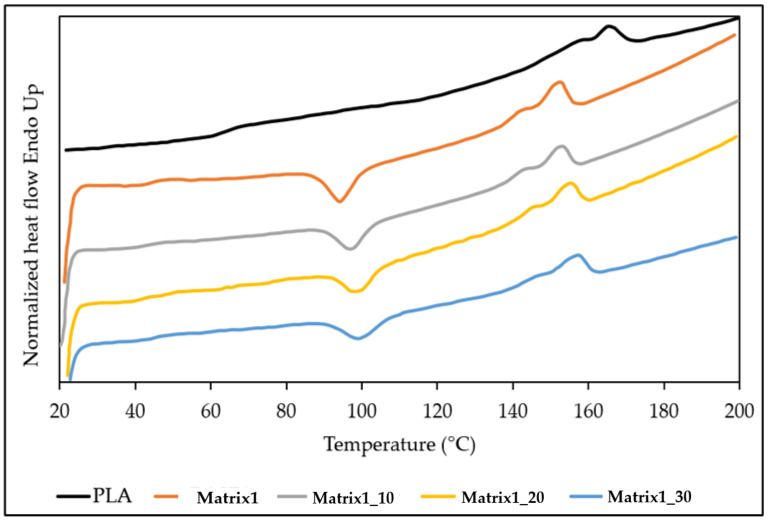
DSC Thermogram of Matrix1 series compared with pure PLA.

DSC analysis was conducted to assess the effect of PS on polymer matrix transitions. Figure 7 shows the DSC curves of the 2nd heating of PLA and Matrix1 series, and Table 4 shows the average values of Xcc (crystallinity percentage), glass transition temperature, T_g_, crystallization temperature and enthalpy, T_c_ and **Δ**h_c_, melting temperature and enthalpy, T_m_ and **Δ**h_m_.

From the comparison between the thermograms of PLA and of Matrix1, it can be noticed that PLA shows a wide event between 90 and 140 °C with a minimum at about 115 °C, attributable to the cold crystallization of the polymer during the 2nd heating. The same exothermic event resulted more evident in the Matrix1 and occurred at a lower temperature (96 °C), indicating a higher crystallization capacity of the sample, due probably to the presence of the PBAT. In fact, PBAT can act as nucleation agent by promoting PLA crystallization [40,41]. Moreover, as also described by Haddar and Qiao [16,19], PS, used as a filler in HDPE-based composites, can promote a slight increase in the crystallinity of HDPE.

As can be observed, similarly to the T_g_, also the melting temperature of Matrix1 is lower than that of neat PLA. In any case, the presence of PS, even at 30 wt.%, did not appreciably modify the characteristic temperatures and relative enthalpies of the Matrix1.

### 3.7. Injection Moulded Pots Production

On the basis of the good results obtained in terms of process-ability, rheological and mechanical properties, Matrix1_20 and Matrix1_30 were selected to carry out the industrial moulding of plant pots at Nuova Pasquini e Bini S.P.A. (Lucca, Italy), a pots moulding company. Matrix1_20 and Matrix1_30 were chosen given their high PS content and, consequently, more economically competitive than filler-free matrix.

**Figure 8:**
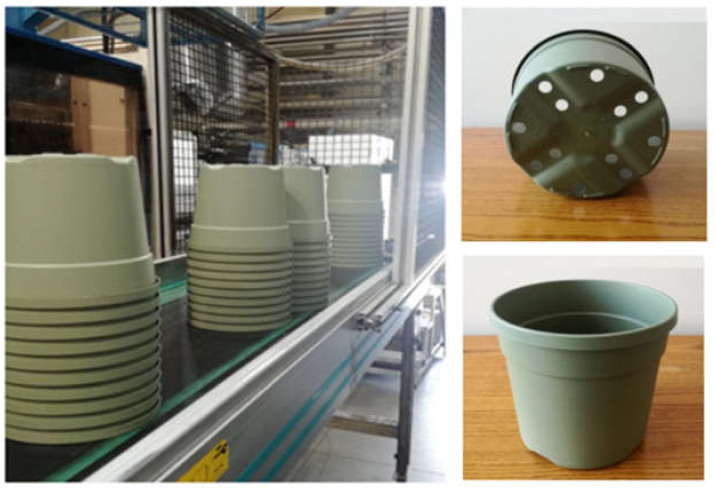
Injection molding pots of Matrix1_30 formulations.

Figure 8 shows photos of the moulded pots (18 cm in diameter) for nurseries. No problem occurred during the moulding phase, the production speed was only reduced due to the longer times required for cooling the mould, reaching 150 pots/h.

The products were flexible and passed the crash test and qualitative analysis conducted on site.

### 3.8. Phytotoxicity Test

**Figure 9:**
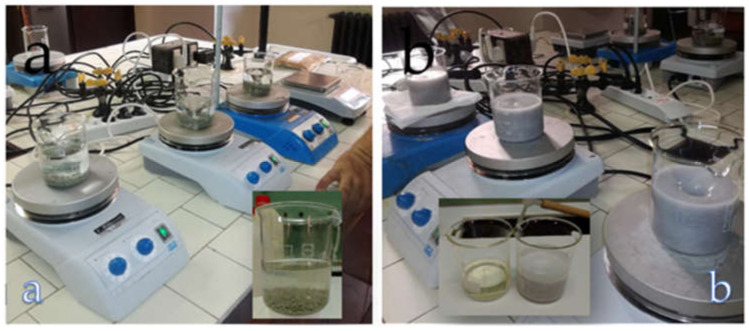
(**a**) Set-up for the preparation of the eluates and (**b**) the eluates obtained from PS and graules containing 30 wt.% PS (Matrix1_30).

In view of the potential use of PS in PLA/PBAT-based composites for the production of items for agriculture such as pots, as investigated in this work, a preliminary phytotoxicity test was performed using eluates extracted from grinded PS and Matrix1_30 granules. Figure 9 shows some photos of set-up used to obtain the eluates and the resultant eluates.

The germination indices obtained, at the end of the incubation period of *L. sativum* seeds, are reported in Table 5. As shown, the GI values were well above the GI threshold value of 60%, considered safe for eluates obtained from compost [42], and well above those found with deionised water (control). These results indicate, therefore, that both the PS as it is and Matrix1_30 granules result not potentially phytotoxic.

It should be noted that in the case of the eluate obtained with PS, roots up to 78 mm long were obtained, evidencing also radicalization in the paper filter.

## 4. Conclusions

In the present work, the possibility of using paper sludge (PS) as filler in bio-composites based on PLA/PBAT and ATBC was investigated. The produced bio-composites showed a good process-ability by extrusion and injection moulding up to 30 wt.% PS, successfully obtaining mould pots for horticulture. Preliminary phytotoxicity tests conducted using *Lepidium sativum* L. seeds showed positive effects of PS eluates on both germination and root elongation. The good process-ability by extrusion and injection moulding of the developed composites up to 30 wt.% PS showed that this waste form paper industry is a real and competitive substitute of calcium carbonate as filler in the production of bio-composites for applications in floriculture and/or horticulture.

## Figures and Tables

**Table 1:** Composition of produced biocomposites (wt %).

Sample	PLA	PBAT	ATBC	CaCO_3_	PS	Total Filler	Ratio PLA/PBAT	Ratio PLA/(PBAT + ATBC)
Matrix1	70	20	10	0	0	0	3.5	2.3
Matrix1_10	63	18	9	0	10	10	3.5	2.3
Matrix1_20	56	16	8	0	20	20	3.5	2.3
Matrix1_30	49	14	7	0	30	30	3.5	2.3
Matrix1c_10	63	18	9	10	0	10	3.5	2.3
Matrix1c_20	56	16	8	10	10	20	3.5	2.3
Matrix1c_30	49	14	7	10	20	30	3.5	2.3

**Table 2:** Results of tensile tests of the biocomposites studied.

Sample	Young’s Modulus (GPa)	Stress at Yield (MPa)	Yield Elongation (%)	Stress at Break (MPa)	Elongation at Break (%)
**Matrix1**	1.5 (0.15 *)	16.1 (1.9 *)	5.6 (1.1 *)	22.2 (0.6 *)	483.2 (10.4 *)
**Matrix1_10**	1.5 (0.27 *)	13.5 (1.6 *)	6.1 (1.8 *)	16.5 (1.6 *)	372.0 (12.8 *)
**Matrix1_20**	1.8 (0.17 *)	13.3 (1.0 *)	5.8 (0.7 *)	12.0 (0.2 *)	119.7 (2.6 *)
**Matrix1_30**	2.0 (0.26 *)	16.2 (1.4 *)	5.2 (1.1 *)	12.3 (0.5 *)	35.6 (5.7 *)
**Matrix1c_10**	1.8 (0.06 *)	25.7 (2.1 *)	4.4 (0.81 *)	26.8 (0.7 *)	445.1 (17.7 *)
**Matrix1c_20**	1.9 (0.04 *)	25.0 (0.5 *)	4.3 (0.75 *)	21.8 (0.1 *)	330.4 (7.3 *)
**Matrix1c_30**	2.1 (0.10 *)	23.8 (0.02 *)	4.7 (0.03 *)	15.3 (0.1 *)	79.0 (31.6 *)

* Standard deviation.

**Table 3:** Charpy Impact Strength values for Matrix1 series.

Sample	Charpy Impact Strength (kJ/m^2^)
**Matrix1**	13.24 (0.99 *)
**Matrix1_10**	5.41 (0.14 *)
**Matrix1_20**	4.74 (0.30 *)
**Matrix1_30**	4.20 (0.45 *)

**Table 4:** Data obtained from DSC measurement.

Sample	T_g_ (°C)	T_c_ (°C)	Δh_c_ (J/g)	T_m_ (°C)	Δh_m_ (J/g)	X cc (%)
**PLA**	64.0	115	-	165.0	17.6	18.9
**Matrix1**	45.0	95.1	12.9	153.3	31.3	28.3
**Matrix1_10**	43.1	97.0	12.4	152.0	32.6	34.5
**Matrix1_20**	44.9	92.7	13.3	155.0	32.4	36.7
**Matrix1_30**	44.6	97.5	13.5	155.6	33.5	43.9

**Table 5:** Germination index (GI) of *L sativum* seeds.

Sample	PS		Matrix1_30
	100% Eluate	50% Eluate	100 % Eluate
**IG (%)**	204	110	123

## Data Availability

The data presented in this study are available on request from the corresponding author.
